# Cardo Polybenzimidazole (PBI-O-PhT) Based Membrane Reinforced with *m*-polybenzimidazole Electrospun Nanofiber Mat for HT-PEM Fuel Cell Applications

**DOI:** 10.3390/membranes12100956

**Published:** 2022-09-29

**Authors:** Igor I. Ponomarev, Kirill M. Skupov, Alexander D. Modestov, Anna A. Lysova, Ivan I. Ponomarev, Elizaveta S. Vtyurina

**Affiliations:** 1 A.N. Nesmeyanov Institute of Organoelement Compounds of Russian Academy of Sciences, Vavilova St., 28, bld. 1, 119334 Moscow, Russia; 2 A.N. Frumkin Institute of Physical Chemistry and Electrochemistry of Russian Academy of Sciences, Leninsky Av., 31, bld. 4., 119071 Moscow, Russia; 3 Kurnakov Institute of General and Inorganic Chemistry, Leninsky Av., 31, 119071 Moscow, Russia

**Keywords:** reinforced membrane, polymer–electrolyte complex, polybenzimidazole, PBI-O-PhT, fuel cell, HT-PEMFC, electrospinning, carbon nanofiber, Pt/CNF, hydrogen crossover

## Abstract

The further development of high temperature polymer electrolyte membrane (HT-PEM) fuel cells largely depends on the improvement of all components of the membrane–electrode assembly (MEA), especially membranes and electrodes. To improve the membrane characteristics, the cardo-polybenzimidazole (PBI-O-PhT)-based polymer electrolyte complex doped with phosphoric acid is reinforced using an electrospun *m*-PBI mat. As a result, the PBI-O-PhT/es-*m*-PBI_net_ · nH_3_PO_4_ reinforced membrane is obtained with hydrogen crossover values (~0.2 mA cm^−2^ atm^−1^), one order of magnitude lower than the one of the initial PBI-O-PhT membrane (~3 mA cm^−2^ atm^−1^) during HT-PEM fuel cell operation with Celtec^®^P1000 electrodes at 180 °C. Just as importantly, the reinforced membrane resistance was very close to the original one (65–75 mΩ cm^2^ compared to ~60 mΩ cm^2^). A stress test that consisted of 20 start–stops, which included cooling to the room temperature and heating back to 180 °C, was applied to the MEAs with the reinforced membrane. More stable operation for the HT-PEM fuel cell was shown when the Celtec^®^P1000 cathode (based on carbon black) was replaced with the carbon nanofiber cathode (based on the pyrolyzed polyacrylonitrile electrospun nanofiber mat). The obtained data confirm the enhanced characteristics of the PBI-O-PhT/es-*m*-PBI_net_ · nH_3_PO_4_ reinforced membrane.

## 1. Introduction

**Figure 1:**
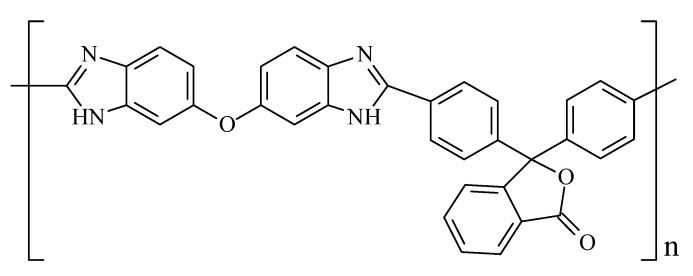
Cardo PBI (PBI-O-PhT) chemical structure.

Polybenzimidazoles (PBIs) are an important class of high-performance heterocyclic polymers that are used as thermo-, heat-, and fire-resistant materials, and as base polymers for proton conducting membranes for fuel cells [1]. A polymer–electrolyte membrane based on PBI is a polymer–electrolyte complex with a large amount of phosphoric acid. The polymer is responsible for the integrity and mechanical properties of the membrane, while phosphoric acid, which is retained by the polymer due to the ionic and hydrogen bonds, provides the proton conductivity. These membranes may work as part of membrane–electrode assembly (MEA) at temperatures above 140 °C, up to 220 °C (unlike Nafion^^®^^-type or similar membranes), providing an excellent performance and greater impurity resistance of the platinum catalyst. The most widely studied PBIs are the commercialized products Celazole^®^ (BASF, Ludwigshafen am Rhein, Germany), Dapozol^®^ (Blue World Technologies, Aalborg, Denmark), and ABPBI Fumapem AM (FuMA-Tech, Bietigheim-Bissingen, Germany) [1,2,3,4,5,6,7,8]. However, the search for new PBI membranes, especially PBI composite proton conducting membranes, remains a challenging task [5]. Cardo PBI (PBI-O-PhT), based on 3,3′,4,4′-tetraaminodiphenyl ether and 4,4′-diphenylphthalide dicarboxylic acid, is a very promising polymer for use as a base polymer for proton conducting membranes (Figure 1).

The synthesis and properties of PBI-O-PhT have been described in detail [9]. MEAs, based on composite or pure PBI-O-PhT membranes, exhibit high performance characteristics, comparable to, and in some cases even exceeding, the commercial ones [10,11,12,13,14]. Among the valuable properties of the PBI-O-PhT membrane doped with phosphoric acid, a high degree of doping that reaches 23 molecules per repeating unit, while maintaining good strength properties has been noted. Other properties include anexcellent proton conductivity (up to 0.1 S cm^−1^), low electrochemical hydrogen crossover (~3 mA cm^−2^) when operating in a high temperature polymer electrolyte membrane (HT-PEM) fuel cell at 180 °C, and a high stability under long-term galvanostatic conditions (>2200 h without degradation) and after multiple start–stop tests. Despite the advantages listed above, the PBI-O-PhT membrane requires further improvement. This becomes even more important when using the latest electrospun carbon-nanofiber (CNF)-based gas-diffusion electrodes (GDEs) Pt/CNF with a higher stability [15,16,17,18,19] compared with the commercial Celtec^®^P1000 (BASF, Ludwigshafen am Rhein, Germany). The necessity to replace carbon-black-based electrodes for HT-PEM fuel cells with more stable ones, for example, based on carbon nanostructured materials, such as CNF, has been extensively discussed in the literature [20,21,22,23,24,25,26,27,28,29]. Earlier, we showed a method to obtain an *m*-PBI electrospun mat (es-*m*-PBI_net_) [17,30,31,32,33,34,35], which was obtained from the commercial *m*-PBI. In order to improve the physical and mechanical properties of the membrane, increase their service life while maintaining high proton conductivity and low gas (hydrogen, oxygen) crossover, and adapt it to a new type of GDE, the PBI-O-PhT membrane needs to be reinforced.

In this study, we report on the possibility of PBI-O-PhT membrane reinforcement with an *m*-PBI electrospun mat (es-*m*-PBI_net_) for the first time, as well as the application of PBI-O-PhT/es-*m*-PBI_net_ · nH_3_PO_4_ resulting in a composite membrane in the HT-PEM fuel cell with the Celtec^®^P1000 and Pt/CNF cathodes.

## 2. Materials and Methods

### 2.1. Preparation of Membranes

#### 2.1.1. PBI-O-PhT Membrane Preparation

The PBI-O-PhT polymer for membrane preparation was synthesized from 3,3′,4,4′-tetraaminodiphenyl ether and 4,4′-diphenylphthalidedicarboxylic acid in the Eaton’s reagent (P_2_O_5_:MeSO_3_H 9:1 wt/wt), according to the procedure described in [9]. The intrinsic viscosity [η] of the solution was determined by extrapolating the reduced viscosity to zero concentration (η_red_ = (t − t_0_)/ct_0_; where t and t_0_ are the flow times of the solution and solvent, respectively) in N-methylpyrrolidone (NMP) at 25 °C, which was 2.02 dL g^−1^ and corresponds to a molecular weight of ~90 kDa [9].

The standard procedure for the PBI-O-PhT film obtaining is described below and includes casting from a PBI-O-PhT polymer solution (5 wt%) on glass plates preliminary heated at 60–80 °C. After solvent evaporation (8–12 h), the films were heat-treated in a vacuum oven at 140 °C for 2 h for additional drying. Then, they were placed in water and were removed from the substrate. For crosslinking, the films were heat-treated at 350 °C for 1 h in an oven with air circulation.

In this study, the PBI-O-PhT membrane was obtained using the Zr procedure [11], in which zirconium (IV) acetylacetonate Zr(acac)_4_ was dissolved in NMP, and then added to the PBI-O-PhT solution to reach 0.075 wt% concentration before casting. The subsequent steps were the same as in the standard procedure. The mechanical properties of the crosslinked film were as follows: tensile strength, σ 110 ± 8 MPa; Young’s modulus, E 1800 ± 70 MPa; and elongation at break, ε 10 ± 3%.

#### 2.1.2. m-es-PBI_net_ Electrospun Mat Preparation

*m*-PBI was synthesized from 3,3′,4,4′-tetraaminobiphenyl and isophthalic acid in polyphosphoric acid, according to [3]. The intrinsic viscosity [η], measured in 98% H_2_SO_4_ at 25 °C, was 0.80 dL g^−1^, which corresponds to a molecular weight of ~30 kDa [36].

*m*-PBI nanofiber mats (*m*-es-PBI_net_) were prepared using the Nanospider^TM^ electrospinning technology (needle-free method from free liquid surface) from *m*-PBI solutions (15 wt%) in the dimethylacetamide/ethanol mixture (9:1 *v*/*v*) on an NS Lab setup (Elmarco, Liberec, Czech Republic). Two types of substrates were used: Al foil and siliconized paper.

The electrospinning onto the Al foil was performed at 22 °C, at a relative humidity of 25%. The electrospinning parameters were as follows: voltage V of +60/−15 kV (spinning electrode/collecting electrode), current I of 0.19–0.20 mA, carriage speed of 3.0 s, in/out air flow of 100/120 m^3^ h^−1^, orifice size of 0.7 mm, and spinning distance of 180 mm.

The electrospinning onto siliconized paper was performed at 22 °C, at a relative humidity of 25%. The electrospinning parameters were as follows: voltage V of +60/−35 kV (spinning electrode/collecting electrode), current I of 0.33–0.34 mA, carriage speed of 4.0 s, in/out air flow of 60/80 m^3^ h^−1^, orifice size of 0.7 mm, and spinning distance of 180 mm. As a result, *m*-PBI nanofiber mats were obtained (Figures S1–S3).

In order to use the *m*-es-PBI_net_ mats as a reinforcing material for the PBI-O-PhT membrane, their heat-treatment or stabilization (350 °C, air) is necessary in order to crosslink *m*-PBI and to make it insoluble in amidic solvents (Figure S4).

#### 2.1.3. PBI-O-PhT/es-m-PBI_net_ Composite Membrane Preparation

In order to obtain the composite membrane, the PBI-O-PhT solution with Zr(acac)_4_ was cast onto a dry crosslinked es-*m*-PBI_net_ nanofiber mat (which was removed from siliconized paper and crosslinked at 350 °C) aligned on a glass substrate. After solvent evaporation, the composite film was heat-treated in the same way as described above for the PBI-O-PhT membrane.

In order to obtain the composite membrane on an aluminum surface, the PBI-O-PhT solution with Zr(acac)_4_ was cast onto an aligned es-*m*-PBI_net_ nanofiber mat located on the aluminum surface just after electrospinning, and was heat-treated on this substrate at 350 °C for crosslinking (Figure S5). The mechanical properties of the crosslinked film were as follows: tensile strength, σ 125 ± 6 MPa; Young’s modulus, E 2800 ± 100 MPa; and elongation at break, ε 8 ± 2%.

#### 2.1.4. Doping with Phosphoric Acid

In order to provide proton conductivity to the membranes and PBI nanofiber mat, the obtained samples were kept for 3 days in phosphoric acid (77 wt%) at 60 °C; then, they were kept for 3 days in phosphoric acid (85 wt%) at room temperature. The resulting PBI-O-PhT/es-*m*-PBI_net_ · nH_3_PO_4_ composite membrane thickness was ~69 ± 5 µm and the PBI-O-PhT membrane thickness was 82 ± 4 µm, with masses ~400% of the undoped ones for both. The electrospun es-*m*-PBI_net_ nanofiber mat thickness was 81 ± 3 µm, with a mass ~800% of the undoped one. The membranes were stored in phosphoric acid (85 wt%) at room temperature.

### 2.2. Proton Conductivity Measurements

The proton conductivity for the phosphoric acid initial PBI-O-PhT, *m*-PBI electrospun mat (es-*m*-PBI_net_), and PBI-O-PhT/es-*m*-PBI_net_ · nH_3_PO_4_ doped composite membrane (S cm^−1^) was performed by electrochemical impedance spectroscopy on a Z500 Pro impedance meter (Elins, Chernogolovka, Russia) in a frequency range of 10–1.5 × 10^6^ Hz in the potentiostatic mode with a sinusoidal signal amplitude of 80 mV, applying graphite electrodes in the two-electrode cell. The samples were doped by *o*-phosphoric acid (conc. 77 wt%) at 60 °C over 3 days. Then, the samples were replaced into *o*-phosphoric acid with a concentration of 85 wt% for 3 days at room temperature. Before the measurements, the samples were wiped with a filter paper to remove excess acid. The proton conductivity was measured at 25–180 °C in steps of 10–15 °C. The sample resistance (*R*) values were calculated at the intercept of the Nyquist plot semicircle with the impedance real axis at a high frequency. The proton conductivity [37,38,39] (σ, S/cm) was calculated according to Equation (1)

(1) $$\sigma =\frac{h}{SR}$$

where *h* is the distance between electrodes (membrane thickness) and *S* is the electrode area.

The activation energy (E_a_) of the conductivity was calculated graphically from the dependence of lgσ from (1000/T) using the Arrhenius Equation (2) [40]

σ = σ_0_ exp (−E_a_/RT) (2)

### 2.3. Gas Permeability Measurements

The gas permeability values for the membranes were obtained according to a previously reported procedure [41]. Briefly, the experiments were carried out in a cell divided into two parts by the membrane in a thermostat. During the experiment, pure hydrogen from a Khimelectronika hydrogen generator (Moscow, Russia) was supplied to one part of the cell and argon was supplied to another part, at a flow rate of 20 mL min^−1^ for both gases. The hydrogen concentration was obtained using a Crystallux-4000M gas chromatograph 4000M (Meta-Chrom, Yoshkar-Ola, Russia) with a heat conductivity detector (current of 30 mA) and packed column (Mole Sieve 5 A; sorbent, 2 m, 30 °C, 20 cm^3^/min, Ar). The membrane gas permeability values *P* (cm^2^ s^−1^) were calculated at 30 and 50 °C according to Formula (3) [42]

(3) $$P=\frac{jL}{C_{L}-C_{R}}$$

where *L* is the membrane thickness (cm), *C_L_* is the average hydrogen volume concentration of the hydrogen in the hydrogen part of the cell (mol cm^−3^), and *C_R_* is the average hydrogen volume concentration in the argon part of the cell (mol cm^−3^). The gas flow across the membrane values *j* (mol cm^−2^ s^−1^) was obtained from Formula (4) [42]

(4) $$j=\frac{CV_{t}}{S}$$

where *C* is the hydrogen concentration (mol cm^−3^) at the outlet of the argon part of the cell (at standard conditions), *V_t_* is the volume flow rate of the carrier gas (cm^3^ s^−1^), and *S* is the active area of the membrane (cm^2^).

### 2.4. Preparation of CNF Electrode

To obtain the CNF electrode, a procedure similar to those that have already been described in our previous studies was employed [14,15,16,17,18,19]. Zirconium (IV) chloride, nickel (II) acetate, polyacrylonitrile (PAN), H_2_[PtCl_6_]·6H_2_O, and DMF (Merck KGaA, Darmstadt, Germany) were used as received. The composite PAN-based carbon nanofiber electrode was obtained using the Nanospider^TM^ electrospinning technology (needle-free method from free liquid surface at a relative humidity of 8%, voltage of 69 kV, and distance between electrodes of 190 mm) on a NS Lab Nanospider^TM^ setup (Elmarco, Liberec, Czech Republic) from a solution containing 3.25 g of PAN (M_w_ 150 kDa), 0.1 g of Vulcan^®^XC72 (Cabot Corporation, Boston, MA, USA) carbon black (~3 wt% relative to PAN), 0.03 g of zirconium (IV) chloride, and 0.40 of nickel (II) acetate well dispersed in 50 mL of DMF for 3 h in an ultrasonic bath. As a result, a composite nanofiber mat was obtained. To obtain the CNF mat, the nanofiber mat was stabilized (350 °C in air) and pyrolyzed (1000 °C under vacuum). The resulting CNF contained 84.7% of C, 2.1% of N, and 0.4% of H, according to the elemental analysis. Platinum was deposited on the CNF mat (mass 12.1 mg, area 6.76 cm^2^) in 10 mL of water containing 23 mg of H_2_[PtCl_6_]·6H_2_O and 0.5 g of HCOOH. The solution with the CNF mat was left for 3 days, then washed with distilled water and dried at 100 °C for 2 h to obtain the Pt/CNF cathode with 8.5 mg of Pt (1.26 mg_Pt_ cm^−2^).

### 2.5. HT-PEM Operation

#### 2.5.1. Fuel Cell Testing

For the membrane and electrode tests, the MEAs were prepared with a working area of 5 cm^2^. The MEAs were placed in a standard Arbin Instruments testing cell (College Station, TX, USA) with two graphite flow field plates. The fuel cell was operated with typical Celtec^®^-P 1000 MEA anode and cathode [43], or using Celtec^®^-P 1000 MEA anode and CNF cathode. Fuel cell operation was carried out at 180 °C. Hydrogen and air were supplied using a G-40 fuel cell test station (Hydrogenics, Mississauga, ON, Canada). The anode was supplied with hydrogen at a rate of 200 mL min^−1^ and the cathode was supplied with air at a rate of 800 mL min^−1^. The gases were supplied without additional humidification. The station was also used to obtain the I–V curves and temperature control, as well as the gas pressure control in the experiments of hydrogen crossover through the membrane. For voltammetry measurements, the fuel cell voltage was scanned at a rate of 5 mV s^−1^ in the cell voltage range of 0.95–0.1 V.

#### 2.5.2. Accelerated Stress-Test

The stress test consisted of 20 repeating cycles. Each cycle comprised heating the HT-PEM fuel cell up to 140 °C without a supply of reagent gases; further heating to 180 °C with the attached gas supply lines and flows of hydrogen and air through the respective cell compounds in the open circuit voltage (OCV) mode; MEA operation at 0.1, 0.2, and 0.4 A cm^−2^ for 30–60 min and then measuring the four consecutive *I*–*V* curves at a rate of 5 mV s^−1^ between OCV and 0.1 V; cooling of the HT-PEM fuel cell to 140 °C with hydrogen and air flowing through the respective compartments in the OCV regime; and cooling the HT-PEM fuel cell to room temperature without the attached gas supply lines.

#### 2.5.3. Membrane Resistance

The measurements of the membrane resistance through plane (mΩ cm^2^) were performed using the method of electrochemical impedance spectroscopy (EIS). The EIS experiments were performed on a PARSTAT 2273 potentiostat-galvanostat-EIS (Prinston Applied Research-AMETEK, Oak Ridge, TN, USA) in open circuit voltage mode or at 0.4 A cm^−2^, applying a sinusoidal current with an amplitude of 20 mA in a frequency range of 50 kHz–0.1 Hz.

#### 2.5.4. Hydrogen Crossover Measurements

Hydrogen crossover during the HT-PEM operation was measured by supplying hydrogen to the MEA gas diffusion anode and nitrogen (99.999% purity) to the gas diffusion cathode. The voltage between electrodes was set to 0.5 V. The overpotential on the hydrogen electrode was negligible. Therefore, the potential of this electrode was very close to the potential of the hydrogen reference electrode. Hydrogen from the hydrogen electrode compartment penetrated the membrane and appeared in the catalyst layer of the counter electrode. At 0.5 V, it was oxidized completely. Thus, the current value measured at these conditions was equal to the flow of hydrogen diffusing through the membrane. Because of low membrane gas permeability, the crossover current was very low. Sometimes, the value was found to be comparable with the oxidation/reduction currents of impurities. The stationary hydrogen crossover current was established within 10–20 min, as a rule. However, in the case of a very low crossover rate, the measurements required a few hours.

### 2.6. Tensile Testing

The mechanical tests of the samples were performed on a 2166 R-5 tensile strength testing machine (Tochpribor, Ivanovo, Russia) in the tension mode. The crosshead speed was 10^−4^ m s^−1^. The mechanical properties (tensile strength, Young’s modulus, and elongation at break) were obtained from the stress–strain curves. The samples with a length of 20 mm and width of 1.5 mm were applied; five experiments were conducted with an accuracy of ~10%.

## 3. Results

### 3.1. Membrane Proton Conductivity and Gas Permeability

**Figure 2:**
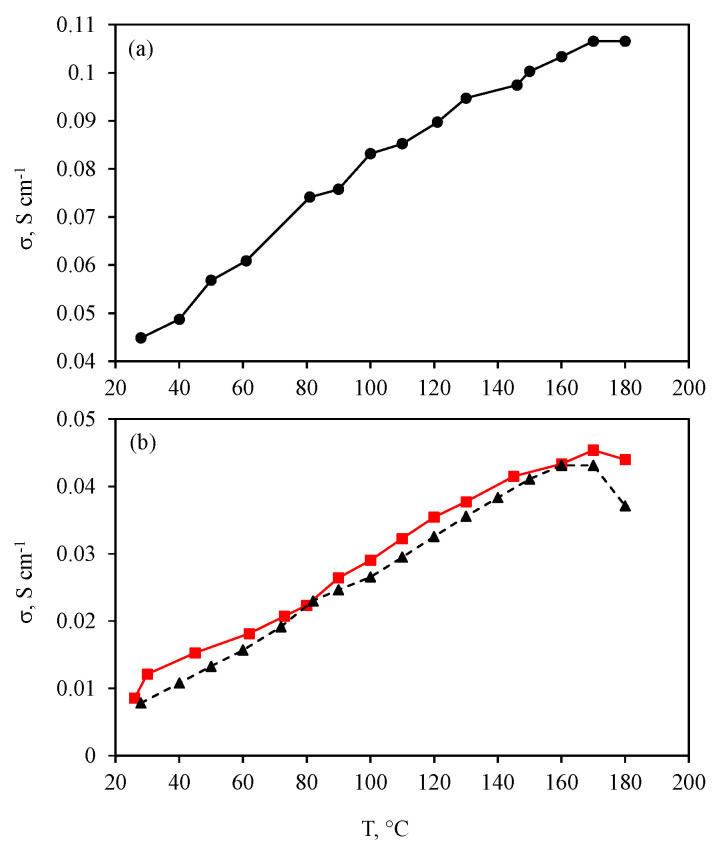
Proton conductivity data for the samples doped with phosphoric acid with an (**a**) electrospun m-PBI mat (es-*m*-PBI_net_), and (**b**) initial PBI-O-PhT membrane (triangles) and composite PBI-O-PhT/es-*m*-PBI_net_ · nH_3_PO_4_ membrane (squares).

Membrane proton conductivity is a very important property of membranes as it allows for making an assessment of the possibility to employ a membrane for fuel cell applications. The proton conductivity data were obtained for the initial membrane and for the composite one (PBI-O-PhT·nH_3_PO_4_ and PBI-O-PhT/es-*m*-PBI_net_ · nH_3_PO_4_, respectively), as well as for the electrospun *m*-PBI mat doped with phosphoric acid (Figure 2a,b).

As can be seen from Figure 2b, the proton conductivity for the initial PBI-O-PhT and composite membrane was in the range of 0.04–0.05 S cm^−1^ at 160 °C. At 180 °C, the proton conductivity value for the initial PBI-O-PhT membrane dropped from 0.043 to 0.037 S cm^−1^. The conductivity activation energy for the initial PBI-O-PhT membrane was 12.8 ± 0.4 kJ mol^−1^. At the same time, the value for the composite membrane remained almost unchanged, at 0.045 and 0.044 S cm^−1^, respectively, and the conductivity activation energy was 10.9 ± 0.3 kJ mol^−1^. These values fit the requirements and were enough for use in HT-PEM applications [5]. The electrospun *m*-PBI doped with phosphoric acid was also tested for proton conductivity to assess its suitability to be used as a reinforcing material (Figure 2a). The proton conductivity value for the doped es-*m*-PBI_net_ reached even higher values of ~0.11 S cm^−1^ at 160–180 °C, and the conductivity activation energy was 6.6 ± 0.3 kJ mol^−1^. This makes it suitable as a reinforcing material for the PBI-O-PhT membrane without the risk of reducing the proton conductivity for the reinforced membrane compared to the initial one.

Hydrogen and oxygen permeabilities were obtained for the undoped composite membrane and for the one doped with phosphoric acid, and are provided in Table 1.

As can be seen from Table 1, all of the values of the hydrogen and oxygen permeability were very low. However, they were slightly higher for the doped composite membrane compared with the undoped one. This can be related to the swelling of the material in phosphoric acid.

### 3.2. HT-PEM Fuel Cell Operation

**Figure 3:**
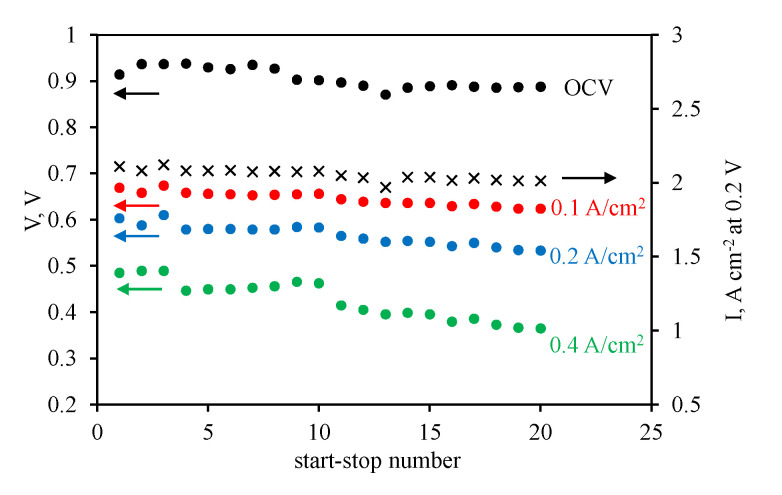
Variation of the open cell voltage and cell voltages measured at 0.1, 0.2, and 0.4 A cm^−2^ variation with start–stop cycle number. The MEA was assembled with the reinforced membrane, Celtec^®^P1000 anode, and Celtec^®^P1000 cathode. The second Y-axis shows the current density measured at 0.2 V in this series of experiments.

For MEAs with the PBI-O-PhT/es-*m*-PBI_net_ · nH_3_PO_4_ reinforced membrane, a stress test consisting of 20 start–stop cycles was performed. The diagram of the voltage values (at a current density of 0 (OCV), 0.1, 0.2, and 0.4 A cm^−2^) and current density values (at a voltage of 0.1 V) after each start–stop cycle for the MEA with Celtec^®^P1000 electrodes are shown in Figure 3.

As can be seen from Figure 3, the usage of the commercial carbon-black-based electrodes Celtec^®^P1000 and reinforced membrane PBI-O-PhT/es-*m*-PBI_net_ in the HT-PEM fuel cell led to a gradual decrease in voltage values at a certain current density with an increase in the number of the start–stop cycles. Particularly, after the stress test, the voltage decreased from 0.715 to 0.684 V at 0.1 A cm^−2^, from 0.669 to 0.624 V at 0.2 A cm^−^^2^, and from 0.603 to 0.533 V at 0.4 A cm^−2^. In addition, the current density decreased from 1.390 to 1.014 A cm^−2^ at 0.2 V.

**Figure 4:**
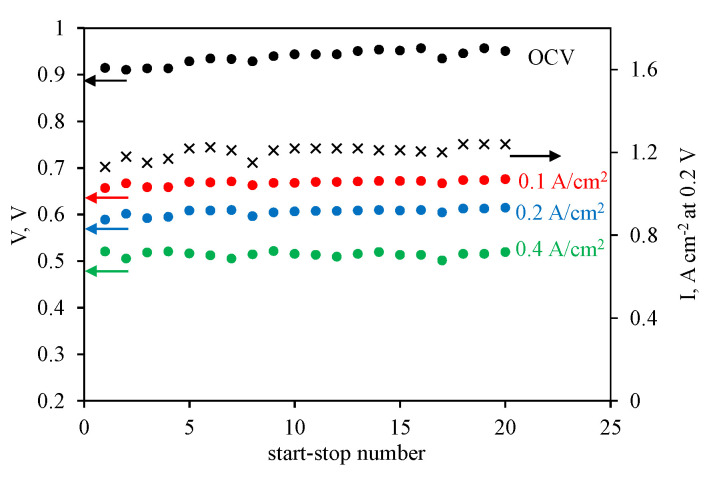
Variation of the open cell voltage and cell voltages measured at 0.1, 0.2, and 0.4 A cm^−2^ variations with a start–stop cycle number. The MEA was assembled with the reinforced membrane, Celtec^®^P1000 anode, and CNF cathode. The second Y-axis shows the current density measured at 0.2 V in this series of experiments.

The MEA consisted of the CNF cathode, and the membrane showed a stable performance in the HT-PEM fuel cell (Figure 4).

Particularly, after the stress tests, the voltage remained almost unchanged or even increased slightly: 0.657 and 0.676 V at 0.1 A cm^−2^, 0.589 and 0.615 V at 0.2 A cm^−2^, and 0.521 and 0.520 V at 0.4 A cm^−2^. The same tendency was found for the current density, namely, 1.13 and 1.24 A cm^−2^ at 0.2 V. Although the values of the voltage at a certain current density were lower before the stress test, they became significantly higher compared with the MEA of the commercial Celtec^®^P1000 electrodes with the same membrane. Examples of the polarization curves before and after the stress tests for the Celtec^®^P1000 and CNF cathodes are shown in Figure S6.

The membrane resistance (R_m_) was studied before and after the stress test using the EIS method under HT-PEM fuel cell operation conditions (180 °C, 0.4 A cm^−2^). It was found that R_m_ did not change after the stress test (Table 2) for MEA with the PBI-O-PhT/es-*m*-PBI_net_ · nH_3_PO_4_ composite membrane. This fact suggests the high stability of the membrane to the stress test. The hydrogen crossover values for MEA with the reinforced composite membrane and Celtec^®^P1000 anode were determined at 180 °C, when nitrogen instead of air was supplied to the cathode at 0.5 V. The crossover values were found to be the same (0.19 mA cm^−2^ atm^−1^) for the MEA with a Celtec^®^P1000 cathode and increased from 0.20 to 0.27 mA cm^−2^ atm^−1^ for the MEA with the CNF cathode (Table 2). These values indicate the insignificant impact of the stress tests on the reinforced composite membrane.

It is necessary to mention that the hydrogen crossover values for the MEAs with composite membrane are very low and one order of magnitude lower than the ones for the initial PBI-O-PhT or commercial *m*-PBI (Celazole^®^). It confirms high barrier properties of the composite membrane which is extremely important for HT-PEM fuel cell operation.

## 4. Discussion

HT-PEM fuel cells usually operate using a PBI membrane [45,46,47,48,49,50]. The PBI-O-PhT membrane was previously shown to be on the same level, or even better in some cases, compared with m-PBI. The composite PBI-O-PhT/es-*m*-PBI_net_ · nH_3_PO_4_ membrane has been shown to be very similar to the initial PBI-O-PhT in terms of the proton conductivity, and is more durable in terms of the mechanical properties. Gas (hydrogen and oxygen) permeability values at 30 and 50 °C were found to be very low (Table 1). This may mean high barrier properties during HT-PEM fuel cell operation.

The MEAs were subjected to stress tests, which included 20 repeated cycles that consisted of heating up to 140 °C, and then further heating to 180 °C for the gas flow (hydrogen and air) in the open circuit voltage (OCV) mode with MEA operation at 0.1–0.4 mA cm^−2^ for 30–60 min, cooling to 140 °C under gas flow in the OCV regime, cooling to the room temperature without the gas flow. For fuel cells operating at high temperatures (150–200 °C), the main reason for degradation is start–stop modes. This is as a result of the compression and expansion of the cell elements, particularly the membrane doped with phosphoric acid, as well as the concentration and dilution of phosphoric acid with water formed during the MEA operation. The other reasons are phosphoric acid redistribution between the membrane and electrode catalytic layers, and degradation of the Pt catalyst and carbon carrier.

The polymer–electrolyte complex PBI-O-PhT/es-*m*-PBI_net_ · nH_3_PO_4_ is an armed composite membrane doped with phosphoric acid that was obtained by reinforcing the PBI-OPht polymer membrane with an *m*-PBI polymer net. The *m*-PBI polymer net was obtained in the form of a nanofiber non-woven mat (es-*m*-PBI_net_) using the electrospinning method from an *m*-PBI solution. The development of this membrane in the form of a polymer–electrolyte complex with phosphoric acid PBI-O-Pht/es-*m*-PBI_net_ · nH_3_PO_4_ for the HT-PEM fuel cell on the polybenzimidazole membrane was primarily aimed at increasing the mechanical strength of the membrane, as well as reducing gas (hydrogen and air) crossover through the membrane while maintaining proper membrane proton conductivity for the fuel cell operation. The choice of es-*m*-PBI_net_ as a reinforcing net material is related to the fact that this material also forms a polymer–electrolyte complex with phosphoric acid. Therefore, no decrease in the membrane proton conductivity was expected, which was proven by the proton conductivity experiments in the previous section. Assumably, the electrospun nanofibers of es-*m*-PBI_net_ possess an increased density compared with the *m*-PBI film, which determines their increased mechanical characteristics. Their higher mechanical strength may allow for thinner membranes to be applied in the fuel cells, which may result in lower membrane electrical resistance values.

**Figure 5:**
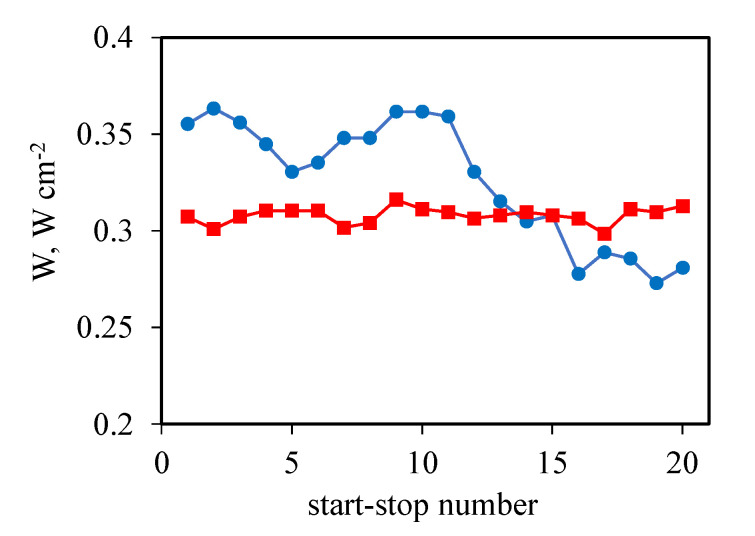
Power density values at 0.8 A cm^−2^ vs. the number of passed start–stop cycles for two MEAs with different cathodes: the Celtec^®^P1000 cathode (circles) and CNF cathode (squares).

As it was shown before, the values of the voltage at a certain current density were lower before the stress test, and they became significantly higher compared with the MEA consisting of the commercial Celtec^®^P1000 electrodes and the same membrane. To show this tendency, the power density values taken from the polarization curves at 0.8 A cm^−2^ vs. the number of passed start–stop cycles for two MEAs with the Celtec^®^P1000 and CNF cathodes, correspondingly, are presented (Figure 5).

**Figure 6:**
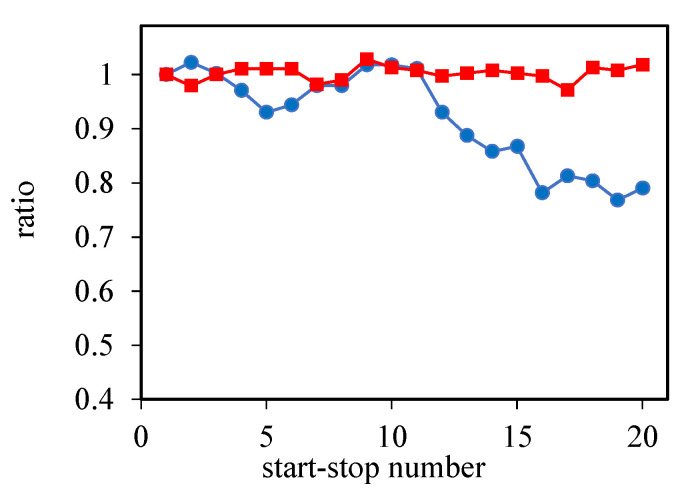
Ratio between the voltage after a given start–stop to the voltage after the first start–stop vs. the number of passed start–stop cycles at 0.8 A cm^−2^ for two MEAs with different cathodes: the Celtec^®^P1000 cathode (circles) and CNF cathode (squares).

In addition, in order to show the performance stability, the obtained data were presented as a graph of the ratio *N* between the voltage after a given start–stop and the voltage after the first start–stop vs. the number of passed start–stop cycles. The data were taken from the I–V curves at 0.8 A cm^2^ (Figure 6).

Thus, the stability of the reinforced membrane PBI-O-PhT/es-*m*-PBI_net_ and CNF electrode, as well as the degradation of the Celtec^®^P1000 electrode after the stress test, are obvious from the obtained data.

In addition, the stability of the reinforced membrane is confirmed by the unchanged membrane resistance (R_m_) obtained before and after the stress tests under HT-PEM fuel cell operation conditions, and by the extremely low hydrogen crossover through the membrane at fuel cell operation conditions when nitrogen was supplied to the cathode at 0.5 V instead of air, before and after the stress tests (Table 2).

## 5. Conclusions

Proton conductivity and gas permeability data show the high barrier properties and suitability of the composite membrane for HT-PEM application. The suitability of the electrospun *m*-PBI mat as a reinforcing material is shown by the high proton conductivity of the *m*-PBI mat doped with phosphoric acid, as well as the very similar proton conductivity values for the initial PBI-O-PhT and reinforced composite PBI O PhT/es-*m*-PBI_net_ membranes.

The stability of the reinforced membrane PBI-O-PhT/es-*m*-PBI_net_ · nH_3_PO_4_ and CNF electrode during HT-PEM fuel cell operation, as well as the degradation of the Celtec^®^P1000 electrode after the stress test are evident from the obtained data. Additionally, the stability of the reinforced PBI-O-PhT/es-*m*-PBI_net_ · nH_3_PO_4_ membrane is confirmed during HT-PEM operation by the extremely low hydrogen crossover and unchanged membrane resistance before and after the stress tests.

## Figures and Tables

**Table 1:** Hydrogen and oxygen permeability values at 30 and 50 °C for the membrane doped with phosphoric acid and for the undoped composite membrane.

Membrane	Gas	P at 30 °C, cm^2^ s^−1^	P at 50 °C, cm^2^ s^−1^
undoped	H_2_	3.4 × 10^−8^	5.1 × 10^−8^
undoped	O_2_	1.1 × 10^−8^	<10^−8^
doped	H_2_	4.1 × 10^−8^	6.1 × 10^−8^
doped	O_2_	2.5 × 10^−8^	3.4 × 10^−8^

**Table 2:** The membrane resistance (R_m_) data (180 °C, 0.4 A cm^−2^) and H_2_ crossover through the membrane (180 °C) in HT-PEM fuel cell operation conditions for MEAs with Celtec^®^P1000 anodes and different cathodes and membranes.

Cathode	Membrane	R_m_, mΩ cm^2^	R_m_, mΩ cm^2^ (After Stress Tests)	H_2_ Crossover, mA cm^−2^ atm^−1^	H_2_ Crossover, mA cm^−2^ atm^−1^ (After Stress Tests)	Ref.
P1000 ^1^	composite	70	70	0.19	0.19	this work
CNF	composite	65	65	0.20	0.27	this work
P1000 ^1^	PBI-O-PhT	~60	n/a	~3	n/a	[11]
P1000 ^1^	*m*-PBI (Celazole^®^)	~55	n/a	4–5	n/a	[44]

^1^ P1000, Celtec^®^P1000 cathode; n/a, not applicable.

## Data Availability

Not applicable.

## Supplementary Materials

The following supporting information can be downloaded at: https://www.mdpi.com/article/10.3390/membranes12100956/s1. Figure S1: SEM of m-PBI electrospun nanofibers on aluminum foil. Figure S2: SEM of stabilized *m*-PBI electrospun nanofibers on aluminum foil. Figure S3: SEM of *m*-PBI electrospun nanofibers on siliconized paper. Figure S4: A photo of stabilized *m*-PBI electrospun nanofibers on aluminum foil. Figure S5: The composite reinforced membrane after crosslinking on aluminum foil. Figure 6: Polarization curves.
