# Supplementary material for: Cardo Polybenzimidazole (PBI-O-PhT) Based Membrane Reinforced with m-polybenzimidazole Electrospun Nanofiber Mat for HT-PEM Fuel Cell Applications
Source: Membranes (Basel). 2022 Sep 29;12(10):956. doi: 10.3390/membranes12100956 (PMC9610054; doi:10.3390/membranes12100956)
Supplement: Supplementary file 1 [file membranes-12-00956-s001.zip › membranes-1935832-supplementary.pdf]

Supplementary Information

Cardo polybenzimidazole (PBI-O-PhT) based membrane reinforced with *m*-polybenzimidazole electrospun nanofiber mat for HT-PEM fuel cell applications

Igor I. Ponomarev [1], Kirill M. Skupov [1,*], Alexander D. Modestov [2], Anna A. Lysova [3], Ivan I. Ponomarev [1] and Elizaveta S. Vtyurina [1]

[1]A.N. Nesmeyanov Institute of Organoelement Compounds of Russian Academy of Sciences, Vavilova St., 28, bld. 1, 119334 Moscow, Russia
[2]A.N. Frumkin Institute of Physical Chemistry and Electrochemistry of Russian Academy of Sciences, Leninsky Av., 31, bld. 4., 119071 Moscow, Russia
[3] Kurnakov Institute of General and Inorganic Chemistry, Leninsky Av., 31, 119071 Moscow, Russia

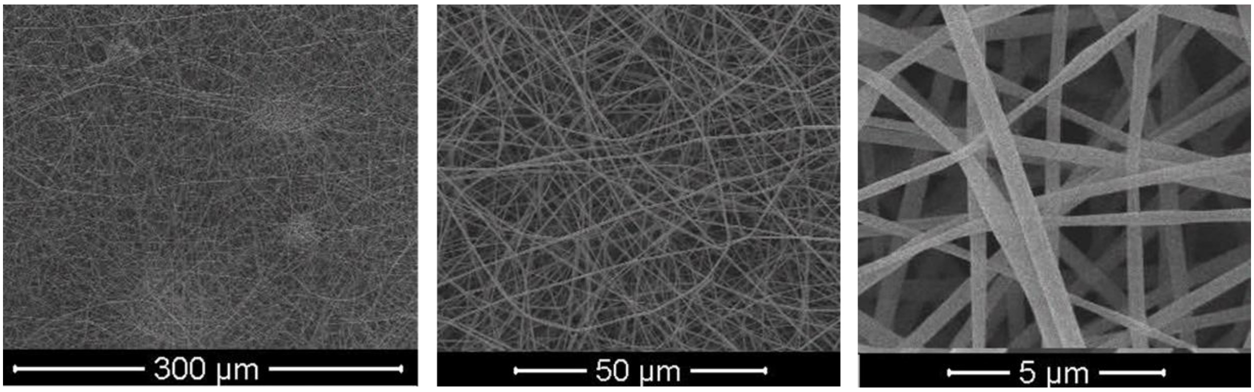

Figure S1. SEM of *m*-PBI electrospun nanofibers on aluminum foil.

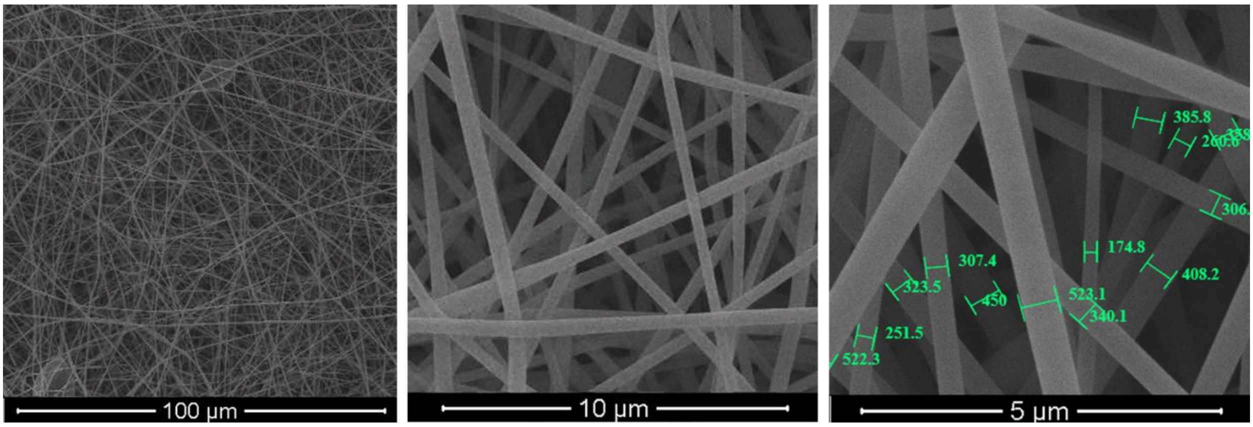

Figure S2. SEM of stabilized *m*-PBI electrospun nanofibers on aluminum foil.

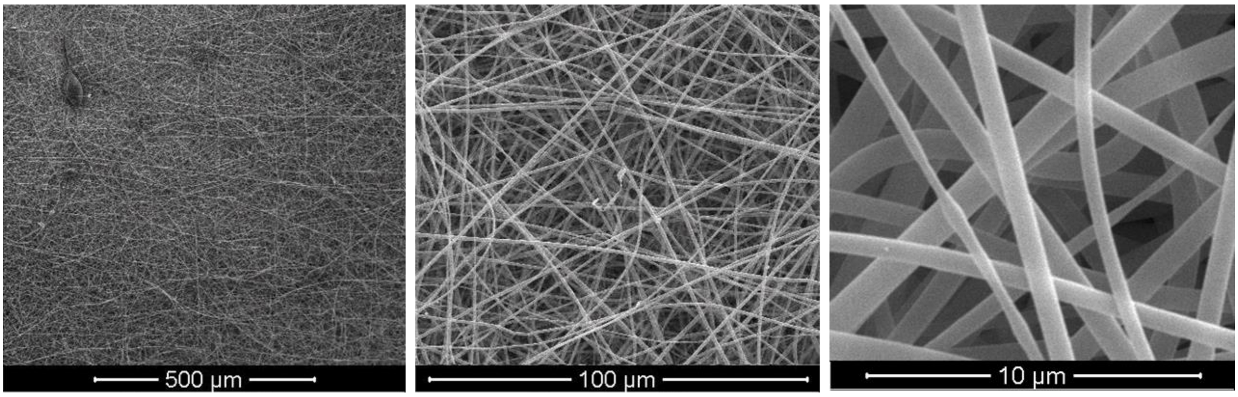

Figure S3. SEM of *m*-PBI electrospun nanofibers on siliconized paper.

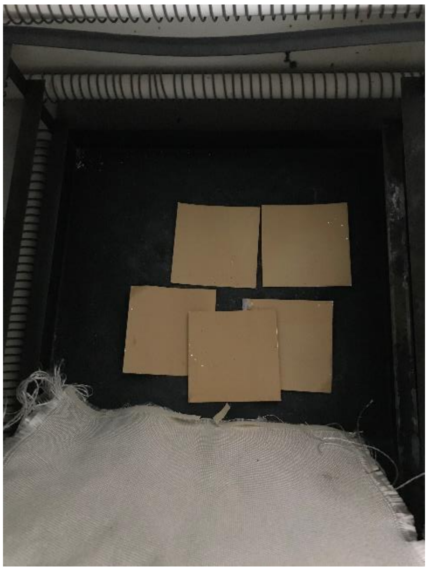

Figure S4. A photo of stabilized *m*-PBI electrospun nanofibers on aluminum foil.

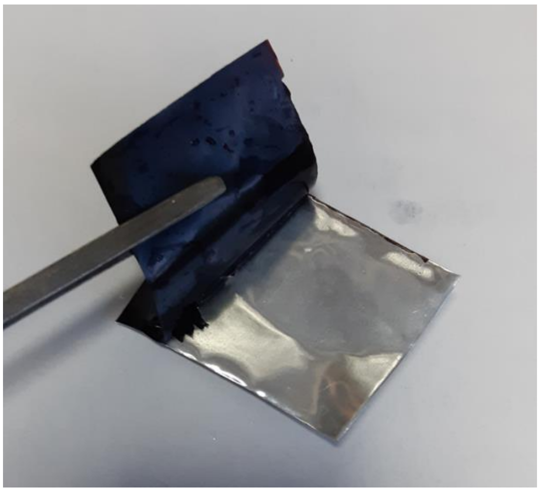

Figure S5. The composite reinforced membrane after crosslinking on aluminum foil.

Polarization curves

polarization curves, 180 °C, cathode - commercial Celtec®P1000 cathode
initial

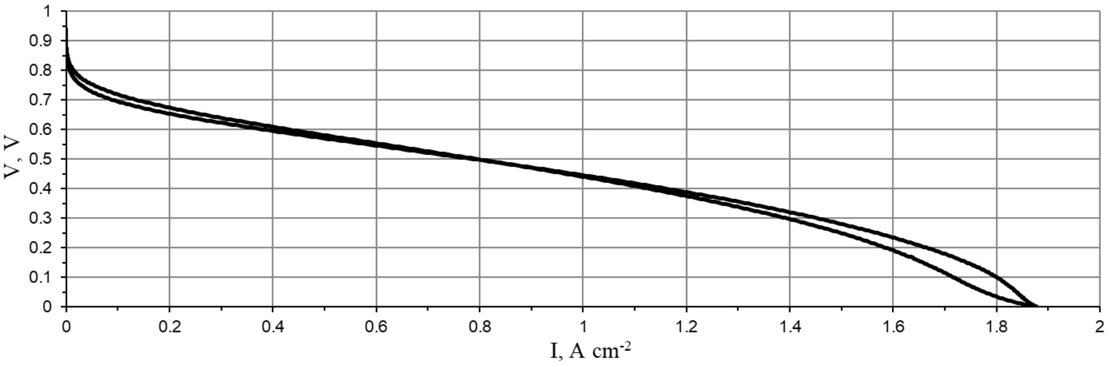

after 20 start-stop cycles

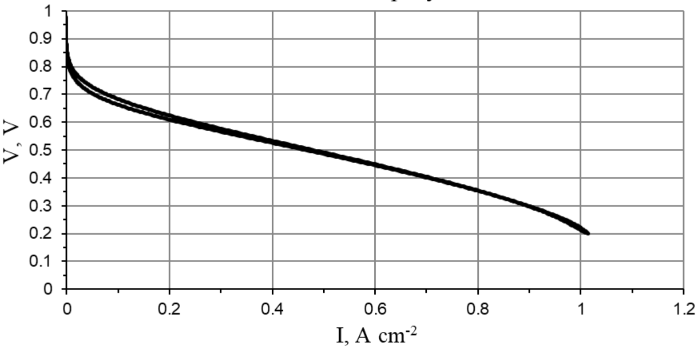

polarization curves, 180 °C, CNF cathode
initial

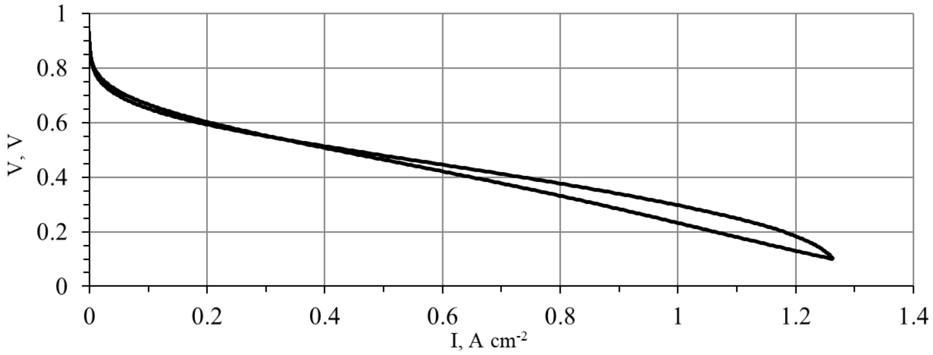

After 20 start-stop cycles

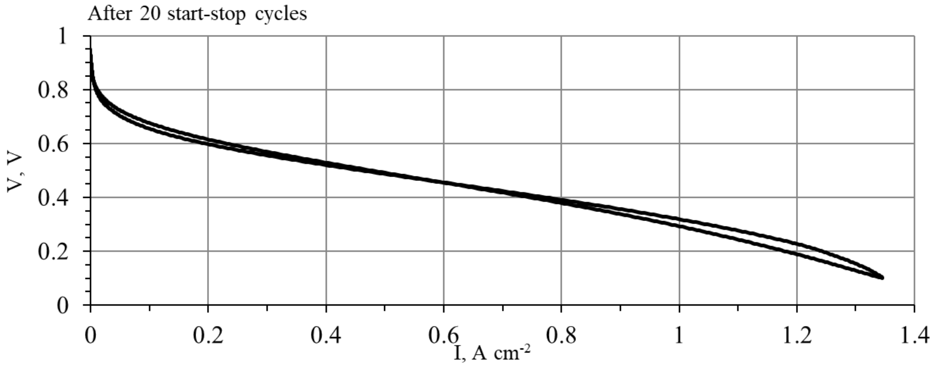

Figure S6. Polarization curves.
